# Wolfram syndrome: A perspective on gene editing as a therapeutic strategy

**DOI:** 10.4103/NRR.NRR-D-25-00835

**Published:** 2025-10-30

**Authors:** Steven Bergmans, Lies De Groef

**Affiliations:** Leuven Brain Institute, Cellular Communication and Neurodegeneration Research Group, Animal Physiology and Neurobiology Division, Department of Biology, KU Leuven, Leuven, Belgium

Wolfram syndrome (WS) is a rare autosomal recessive disease characterized by the development of diabetes insipidus, diabetes mellitus, optic atrophy, and deafness (often referred to as DIDMOAD), and overall severe neurodegenerative fallback. The global prevalence of this disease is estimated at 1 in 770,000 (Lee et al., 2023). It is most commonly caused by biallelic (point)mutations in the Wolframin endoplasmic reticulum (ER) transmembrane glycoprotein (*WFS1*) gene (in case of WS type 1), but mutations in the CDGSH Iron Sulfur Domain 2 (*CISD2*) are also linked to WS (type 2). The latter, however, often present with less severe pathological manifestations (Lee et al., 2023). *WFS1* is located on chromosome 4p16.1 and spans over 33 kilobases. Many mutation variants have been identified in *WFS1*, encompassing missense, nonsense, and frameshift mutations. These mutations are spread across the coding region of *WFS1*, but certain regions, such as exon 8, the largest exon, appear particularly mutation-prone and associated with the classical WS type 1 phenotype (Lee et al., 2023). Wolframin, the protein encoded by *WFS1*, is essential for ER calcium homeostasis, regulation of the unfolded protein response, and ensuring proper ER-mitochondrial communication. Mutations in *WFS1* that result in a loss-of-function, consequently lead to chronic ER stress and activation of apoptotic pathways, particularly in metabolically demanding cells such as pancreatic β-cells and neurons. As such, a spectrum of clinical manifestations arises in WS patients (Lee et al., 2023). The disease typically manifests during early childhood, beginning with diabetes and followed by progressive optic nerve atrophy, hearing loss, and various neurological and urological complications. Other symptoms may include psychiatric manifestations, sleep disturbances, ataxia, and cognitive decline. Life expectancy is significantly reduced, often due to brainstem atrophy and respiratory failure in the third to fourth decade of life (Lee et al., 2023).

Rodent and induced-pluripotent stem cell models have been instrumental in elucidating the pathophysiology of WS, extensively reviewed elsewhere (Morikawa et al., 2024). A first generation of rodent WS models, notably the Wfs1 exon 5 deletion rat and Wfs1 exon 8 deletion mouse, led to the first insights into reduced β-cell mass and progressive glucose intolerance in WS, as well as neurological manifestations such as optic nerve atrophy, cerebellar degeneration, and brainstem atrophy (Morikawa et al., 2024). In recent years, several point mutation mouse models have been developed to better recapitulate specific human genotypes. For instance, the *Wfs1 c.2590G>A p.E846K* knock-in mouse, which models a missense mutation identified in patients with severe vestibular dysfunction, demonstrates sensorineural hearing loss and behavioral abnormalities akin to patients (Morikawa et al., 2024). Finally, several patient-derived induced pluripotent stem cell models have been used to study WS pathology, in β-cell islets, neurons, and oligodendroglia (Kitamura et al., 2022; Zatyka et al., 2023; Ahuja et al., 2024). Collectively, these experimental models have revealed that Wolframin plays a critical role in calcium signaling between the ER and mitochondria, influencing cell survival pathways, mitochondrial integrity, and stress responses. Dysregulation of the unfolded protein response, increased susceptibility to oxidative stress, and mitochondrial fragmentation are hallmark features of these models. Together, these intracellular pathological manifestations lead to apoptosis of β-cells in the pancreas, resulting in the diabetic phenotype, and to neuronal dysfunction and loss, underlying neurological deficits. Importantly, models replicating specific human point mutations offer the most translational insight, paving the way for genotype-specific therapeutic development and for testing targeted gene therapies.

CRISPR (clustered regularly interspaced short palindromic repeats)-based genome editing tools offer a promising route for precise correction of pathogenic point mutations without introducing double-stranded DNA breaks through prime or single nucleotide base editing. These gene editors, such as adenine base editors and cytosine base editors, can efficiently convert A•T to G•C or C•G to T•A base pairs, respectively, in a targeted and predictable fashion (Komor et al., 2016). The enormous potential of this technique as a curative therapy for rare, monogenic diseases such as WS was recently reinforced by the successful application of base editing for Stargardt disease, an inherited retinal disorder caused primarily by mutations in the adenosine triphosphate binding cassette subfamily A member 4 (*ABCA4*) gene (Muller et al., 2025). Dysfunction of ABCA4 leads to the toxic accumulation of bisretinoid in photoreceptors in the retina, resulting in progressive vision loss. In this study, a dual adeno-associated virus system encoding a split-intein adenine base editor was used to correct the common *ABCA4 c.5882G>A* mutation (Muller et al., 2025). The therapy demonstrated high editing efficiency in human retinal explants and retinal pigment epithelial cells in vitro, and in vivo correction was achieved in mouse models and non-human primates after subretinal injection. Editing efficiencies in vivo reached up to 75% in cone photoreceptors and 87% in retinal pigment epithelium cells, with no detectable off-target editing (Muller et al., 2025), highlighting the precision and potential of this approach for hereditary monogenic disorders.

Given that many *WFS1* mutations are single-nucleotide variants, gene editing represents a promising therapeutic strategy for WS patients. A recent study in human induced-pluripotent stem cell-derived β-cells obtained from WS patients demonstrated the technical feasibility of allele-specific corrections using base editing. Using adenine base editors, researchers corrected the disease-causing mutations, restoring normal transcript and protein levels while ameliorating disease-associated phenotypes (Maxwell et al., 2020; Nami et al., 2021). Indeed, in these edited β-cells, insulin secretion in response to glucose was significantly improved, and markers of ER stress, such as CHOP, were reduced (Maxwell et al., 2020). This *in vitro* proof-of-concept underscores the feasibility of base editing for *WFS1*-associated mutations. However, while in theory the modularity of this approach allows it to be used for virtually any WS mutation, i.e., gRNA can be designed for specific mutations, the large diversity and relatively low allele frequency of mutations in WS would require a vast bibliography of gRNAs. Thus, the ultra-rare nature of WS and its mutational heterogeneity complicates large-scale development and discourages investment from pharmaceutical companies driven by market potential. Nonetheless, for patients with well-characterized point mutations and severe phenotypes, base editing offers a highly personalized, potentially curative treatment strategy.

Unlike DNA editing, RNA editing involves transient and reversible modifications of RNA transcripts, potentially circumventing issues related to genomic permanence and off-target effects. Adenosine deaminases acting on RNA can be harnessed to convert adenosine to inosine (read as guanosine by the ribosome) and enable correction of G>A pathogenic mutations at the mRNA level. Precedent for this approach has again been set by emerging RNA editing therapies for Stargardt disease. Early-stage trials have shown restoration of functional protein levels of ABCA4 in photoreceptors and reduced degeneration markers in the outer retina (Liou et al., 2024). However, this approach presents the same limitation as the nucleotide base editors at the genomic level; it must be completely tailored to a specific point mutation and thus only serves specific subgroups of patients. An addition to base editing is RNA exon editing. This approach involves targeted modification of RNA splice sites or exonic sequences to restore correct exon inclusion, exclusion, or frame preservation. In the context of WS, RNA exon editing strategies could theoretically bypass premature stop codons or frameshift mutations located within specific exons or even replace a complete exon in which mutations occur, thereby restoring functional protein expression. This strategy is particularly attractive for diseases with diverse mutation spectra like WS, and could be a game-changer in making personalized medicine accessible for genetically heterogeneous patient populations. As the majority of mutations leading to the development of WS are localized in exon 8, this would be a potential broad-based therapy, curative for a large cohort of patients. Furthermore, the reversibility of RNA editing may also offer safety advantages in pediatric and long-term use settings, such as WS, where off-target consequences must be minimized.

**Figure 1:**
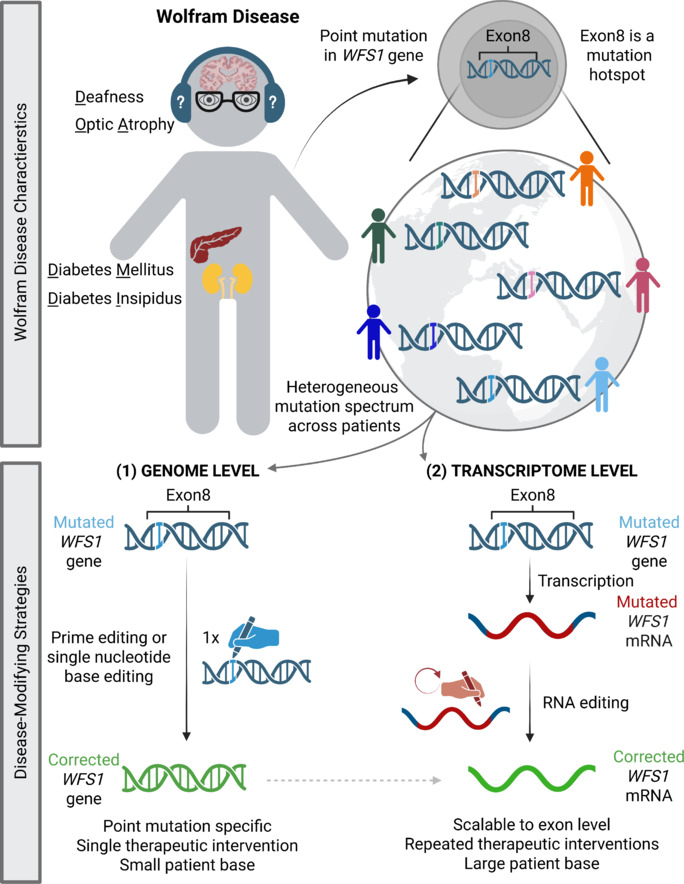
Wolfram disease and the potential of nucleic acid editing strategies as disease-modifying therapies. Wolfram Syndrome (WS) is a rare autosomal recessive disorder characterized by DIDMOAD (Diabetes Insipidus, Diabetes Mellitus, Optic Atrophy, and Deafness) and progressive neurological decline. It is most commonly caused by biallelic point mutations in the *WFS1* gene, with exon 8 being a known mutation hotspot. A wide mutational spectrum exists across WS patients, complicating the development of scalable gene editing therapies. Shown are two potential disease-modifying approaches: (1) DNA editing using prime or base editors, enabling one-time correction of specific point mutations but applicable to a limited patient subgroup; (2) RNA editing, enabling replacement of a full exon, e.g., exon 8, with the potential of treating a broader patient population, though via repeated administrations. Created with BioRender.com.

WS remains an uncurable disorder with devastating consequences for affected individuals. The emergence of rodent models with specific point mutations, however, continues to enhance our understanding of genotype-phenotype correlations and offers new opportunities to develop innovative, personalized treatments. Additionally, the recent development of novel powerful tools for genome and transcriptome engineering offers hope to modify disease onset and progression (**Figure 1**). While CRISPR-derived gene editing offers mutation-specific precision, RNA editing emerges as a broader solution with the potential to address the needs of a more inclusive patient population. As our molecular toolbox expands, longstanding obstacles in rare disease therapy are being dismantled in favor of targeted, effective, and scalable interventions.

*Research into Wolfram syndrome in the De Groef team has been supported by the Eye Hope Foundation (Belgium), Wolfram UK (UK) and The Snow Foundation (USA).*
